# The Influence of Voice Gender and Hearer Gender on Voice‐Related Distress: Examining Gendered Interpersonal Dyads in Voice Hearing

**DOI:** 10.1002/cpp.70323

**Published:** 2026-08-31

**Authors:** Lucas Cabezas Klikovac, Mark Hayward

**Affiliations:** ^1^ School of Psychology University of Sussex Brighton UK; ^2^ Research & Development Department Sussex Partnership NHS Foundation Trust Hove UK

**Keywords:** distress, gender, interpersonal dynamics, voice hearing

## Abstract

**Background:**

Voice‐related distress has traditionally been explained by voice content and severity; however, emerging research highlights the importance of relational, social rank and gendered dynamics. This study examined whether the gender of the hearer and perceived voice gender interacted to influence distress and whether specific hearer gender–voice gender configurations were associated with heightened distress.

**Method:**

Assessments within routine clinical practice were completed by 347 adults referred to a UK specialist clinic for distressing voices between 2016 and 2024. Participants provided data for gender (self and main voice) and voice‐related distress. Hypotheses were tested in relation to perceived voice gender, the pairing of hearer gender and voice gender and associated distress.

**Results:**

Male voices were significantly more common, and men were more likely to hear male voices than women. Women who heard male voices reported the highest levels of distress.

**Conclusions:**

Voice‐related distress appears to be influenced by gendered interpersonal dynamics. Women who hear male voices may be especially vulnerable and could benefit from more targeted, relationally informed support in clinical settings.

## 1 Introduction

Auditory hallucinations, or ‘voices’, are a core symptom of psychotic disorders and are also reported across many other psychiatric diagnoses (Waters and Fernyhough 2017). Voices are increasingly recognized as interpersonal agents with intentions, often appraised as dominant others, triggering patterns of subordination and entrapment (Birchwood et al. 2004). These appraisals are rooted in long‐standing relational patterns, shaped by the hearer's experiences of power and trauma (Bell 2013), and may be understood through evolved social mentality systems for interpreting and responding to interpersonal signals (Gilbert 2020). This perspective has informed a range of relational therapies that aim to change how hearers relate to their voices (see Thomas et al. 2025).

In the context of these relational and social rank influences, gender can signal relational meaning, trigger learned responses, and influence how voices are appraised (Bell 2013). Understanding these gendered identities is therefore critical to exploring why some voices are more distressing than others and how this distress may manifest differently across hearers. Süssenbacher‐Kessler et al. (2021) found that hearers were more likely to perceive their dominant voice to be male. This was particularly the case for men, whilst women reported higher levels of voice‐related distress. Legg and Gilbert (2006) found that voices embody patterns of dominance and submission through insults, warnings and commands, with male voices frequently adopting a shaming, hierarchical tone. This is supported by evolutionary accounts of social competition, where derogatory signals function to regulate social rank (Buss and Dedden 1990), and with Gilbert's (2020) social mentality framework, whereby the gendered characteristics of voices may shape perceptions of social rank threat. McCarthy‐Jones et al. (2015) reported that male voices heard by women were often perceived as coercive or authoritarian, reflecting internalized associations with male control or punishment.

The understandings described above have largely emerged from small or nonclinical samples, limiting the robustness of their conclusions. The aim of the study was to strengthen the literature by testing the following hypotheses using an ecologically valid clinical sample:

Hypothesis 1 *Main voices are more often perceived to be male than female*.

Hypothesis 2 *Men are more likely than women to report a male main voice*.

Hypothesis 3 *Women who hear male voices will report greater distress than other combinations of hearer gender‐voice gender*.

## 2 Methods

### 2.1 Design

This study was a cross‐sectional secondary analysis of routinely collected clinical data extracted from digital assessment records. To explore potential gendered interpersonal dynamics, participants were grouped into four hearer gender–voice gender dyads:

- M‐M: Male participant hearing a male voice
- F‐M: Female participant hearing a male voice
- M‐F: Male participant hearing a female voice
- F‐F: Female participant hearing a female voice

### 2.2 Participants

This study was conducted within the Sussex Voices Clinic (SVC), a specialist service for the psychological treatment of distressing voices within the Secondary Care Adult Services of the UK's National Health Service (NHS). Following referral to and assessment over an 8‐year period within SVC, data were routinely collected by SVC staff as part of standard clinical assessment procedures and included information on demographic characteristics and the primary distressing voice at the time of assessment, including its perceived gender, where reported. Data were included if a patient:

1. provided informed and written consent for the use of their anonymized data for audit and service evaluation projects,
2. offered a clear description of a main voice with a perceived gender,
3. reported their own gender identity.

No additional exclusion criteria were applied, reflecting the aim of analysing routinely collected clinical data from all eligible voice‐hearing individuals within the service.

### 2.3 Ethics Considerations

NHS Research Ethics Committee approval was not required for the study as it was a service evaluation of routine practice within a clinical service. This study received ethical approval from the School of Psychology at the University of Sussex, and the NHS Trust's Quality Improvement Support Team and Research Governance Team.

### 2.4 Materials

Voice‐related distress was assessed with the 4‐item Emotional Impact subscale of the Hamilton. Program for Schizophrenia Voices Questionnaire (HPSVQ; Van Lieshout and Goldberg 2007).

The HPSVQ is a nine‐item self‐report measure with two subscales: a four‐item emotional impact subscale and a five‐item physical impact subscale. Items are scored on a 5‐point rating scale, with higher scores indicating increased severity. The HPSVQ has strong criterion validity, test–retest reliability, high factor loadings and cross‐cultural applicability (Berry et al. 2021).

### 2.5 Analysis

All analyses were conducted in R version 4.4.3. To test Hypothesis 1, a chi‐square goodness‐of‐fit test was used to compare the observed distribution of reported main voice gender (male, female) against a uniform distribution, assessing whether certain voice genders were reported significantly more frequently than others. To test Hypothesis 2, a chi‐square test of independence was conducted. To test Hypothesis 3, a bootstrapped one‐way ANOVA using 20% trimmed means was conducted. This robust approach accounted for potential violations of normality and heteroscedasticity (Field et al. 2012). The four hearer gender–voice gender dyads were used as the grouping variable in the analysis. Post hoc pairwise comparisons were carried out using bootstrap‐adjusted tests to explore significant group differences.

All tests were conducted using a significance threshold of *α* = 0.05 (two‐tailed), and statistical significance was interpreted in line with the a priori hypotheses. No corrections for multiple comparisons were applied, as the analyses were targeted and theory driven. Missing data were removed from each analysis on a case‐by‐case basis using listwise deletion, such that participants were excluded from specific analyses only if they lacked data required for that test.

## 3 Results

### 3.1 Participant Characteristics

A total of 391 eligible patients were assessed between January 2016 and December 2024. Following data cleaning, five patients were excluded due to missing gender data, and one patient was excluded for missing main voice gender data. An additional three patients who selected ‘Other’ as their gender were excluded due to insufficient sample size for group‐level comparisons, and 35 patients who reported a ‘Both’ for voice gender were excluded because this category could not be incorporated into binary gender analyses. The final sample comprised 347 participants (*N* = female 210, *N* = male 137).

The majority of participants identified as White British (*n* = 300, 86.5%). Diagnoses were varied, with the largest groups being psychosis (*n* = 125, 36%), Emotionally Unstable Personality Disorder (EUPD) (*n* = 76, 21.9%) and mood disorders (*n* = 66; 19%).

### 3.2 Hypothesis Testing

#### 3.2.1 Main Voice Gender Distribution (Hypothesis 1)

A chi‐square test for goodness‐of‐fit was conducted on main voice gender frequencies (*N* = 347). A significant deviation from an even distribution was observed, *χ* ^2^(1, *N* = 347) = 46.48, *p* < 0.001. A male main voice was most frequently reported (68.3%; *N* = 237), compared with a female main voice (31.7%; *N* = 110).

#### 3.2.2 Association Between Participant and Main Voice Gender (Hypothesis 2)

A chi square test of independence was conducted. A significant association was found between hearer gender and voice gender, *χ* ^2^(1, *N* = 347) = 7.93, *p* = 0.005. Males were more likely to report a male main voice (77.4%) than females (62.4%), while females were more likely to report a female main voice (37.6%) than males (22.6%).

#### 3.2.3 Voice‐Related Distress by Participant Gender‐Voice Gender Group (Hypothesis 3)

A robust bootstrapped one‐way ANOVA was conducted on HPSVQ emotional impact subscale scores and revealed a statistically significant group difference with moderate effect size, *F*(3, 339) = 5.20, *p* = 0.005, *η* ^2^ = 0.09, *d* = 0.30. The groups were as follows: M‐M (*N* = 103), F‐M (*N* = 131), M‐F (*N* = 31), F‐F (*N* = 78). Mean Distress was highest in the F‐M group (M = 13.1, SD = 12.75), followed by F‐F (M = 12.6, SD = 2.64), M‐M (M = 11.8, SD = 3.25), and M‐F (M = 11.0, SD = 3.93). Post hoc comparisons using 20% trimmed means showed that participants in the F‐M group reported significantly greater distress than those in the M‐M group, *p* = 0.007. No other pairwise comparisons were statistically significant (*p*s > 0.05).

## 4 Discussion

The findings from this study both corroborate and extend prior research, offering further evidence that gendered interpersonal dynamics are central to voice‐hearing experiences.

First, male voices were significantly more common than female voices, replicating earlier findings (Süssenbacher‐Kessler et al. 2021). Approximately two‐thirds of participants identified their main voice as male. Second, participant gender was significantly associated with the gender of the main voice, partially replicating earlier findings (Süssenbacher‐Kessler et al. 2021; Legg and Gilbert 2006). Male participants were more likely to report a male main voice, whereas female participants reported a balanced profile of male and female main voices, although male voices still predominated. Finally, women hearing a male main voice experienced significantly greater distress than men hearing a male main voice, with no significant differences across other dyads. This dyad‐specific effect refines prior findings that women experience greater overall distress (Süssenbacher‐Kessler et al. 2021), suggesting distress is linked to specific gendered relational configurations rather than hearer gender alone (McCarthy‐Jones et al. 2015). Consistent with this finding, perceived voice gender may represent an important feature of the broader interpersonal meaning system through which threat, power and social evaluation are experienced (Legg and Gilbert 2006). Within this framework, gender may shape not only the perceived identity of the voice but also the relational meaning attributed to it, particularly where voices activate pre‐existing schemas related to threat, shame, powerlessness or subordination (Bell 2013; Gilbert 2020).

Clinically, these results suggest that gender should be considered not only as a demographic variable but as a relational lens for understanding voice‐related distress. Routine assessment could include perceived voice gender, followed by exploration of its interpersonal significance. In cases where voices reflect past trauma or evoke feelings of subordination, especially in F‐M dyads, clinicians may benefit from using formulation approaches that integrate gendered relational meaning. Interventions that directly target these dynamics, such as the development of assertive responding (Schlier et al. 2021) and compassion‐focused approaches that aim to reduce threat‐based responding by fostering caring interpersonal mentalities (Heriot‐Maitland et al. 2019; Leach et al. 2024), may be particularly beneficial for this patient group.

Several limitations should be acknowledged when interpreting these findings. The analyses were based on data collected at a single point in time, limiting the ability to draw causal inferences. Additionally, where individuals reported multiple voices, analyses focused on the primary distressing voice identified. This reflects clinical relevance but may not capture variation in voices and voice characteristics across contexts. Diagnostic differences were not controlled for and given the higher prevalence of EUPD among women and the elevated levels of voice‐related distress reported by this patient group, these factors may have contributed to gender effects (Slotema et al. 2012). Other key variables such as trauma history, medication status, and illness duration were not systematically assessed, limiting the ability to account for alternative explanations. The exclusion of nonbinary and gender‐diverse participants due to small subgroup sizes further narrows the scope, restricting the findings to binary gender‐conforming individuals. Moreover, all participants were drawn from a single specialist UK clinical service. While this lends real‐world clinical relevance, local demographics, help‐seeking patterns, and specialist voice hearing service structures may limit wider applicability, particularly across international or nonspecialist contexts.

## Conflicts of Interest

The authors declare no conflicts of interest.

## Data Availability

The data that support the findings of this study are available on request from the corresponding author. The data are not publicly available due to their containing information that could compromise the privacy of the patients.
